# Webuye Health and Demographic Surveillance Systems Baseline Survey of Soil-Transmitted Helminths and Intestinal Protozoa among Children up to Five Years

**DOI:** 10.1155/2013/734562

**Published:** 2013-02-26

**Authors:** A. A. Obala, C. J. Simiyu, D. O. Odhiambo, V. Nanyu, P. Chege, R. Downing, E. Mwaliko, A. W. Mwangi, D. Menya, D. Chelagat, H. D. N. Nyamogoba, P. O. Ayuo, W. P. O'Meara, M. Twagirumukiza, D. Vandenbroek, B. B. O. Otsyula, J. de Maeseneer

**Affiliations:** ^1^School of Medicine, Moi University, Kenya; ^2^School of Public Health, Moi University, Kenya; ^3^School of Nursing, Moi University, Kenya; ^4^Duke University, USA; ^5^Ghent University, Belgium

## Abstract

*Background*. The intestinal parasitic infections (IPIs) are globally endemic, and they constitute the greatest cause of illness and disease worldwide. Transmission of IPIs occurs as a result of inadequate sanitation, inaccessibility to potable water, and poor living conditions. *Objectives*. To determine a baseline prevalence of IPIs among children of five years and below at Webuye Health and Demographic Surveillance (HDSS) area in western Kenya. *Methods*. Cross-sectional survey was used to collect data. Direct saline and formal-ether-sedimentation techniques were used to process the specimens. Descriptive and inferential statistics such as Chi-square statistics were used to analyze the data. *Results.* A prevalence of 52.3% (417/797) was obtained with the male child slightly more infected than the female (53.5% versus 51%), but this was not significant (*χ* ^2^ = 0.482, *P* > 0.05). *Giardia lamblia* and *Entamoeba histolytica* were the most common pathogenic IPIs with a prevalence of 26.1% (208/797) and 11.2% (89/797), respectively. Soil-transmitted helminths (STHs) were less common with a prevalence of 4.8% (38/797), 3.8% (30/797), and 0.13% (1/797) for *Ascaris lumbricoides*, hookworms, and *Trichuris trichiura*, respectively. *Conclusions*. *Giardia lamblia* and *E. histolytica* were the most prevalent pathogenic intestinal protozoa, while STHs were less common. Community-based health promotion techniques are recommended for controlling these parasites.

## 1. Introduction

It is estimated that approximately a billion people in developing countries of the sub-Saharan Africa, Asia, and the Americas are infected with one or more helminths [1]. About 300 million people are severely ill with intestinal parasitic infections (IPIs), out of which, approximately 50% are school-age children [2]. The IPIs are globally endemic and are responsible for the greatest worldwide cause of illnesses and disease [3, 4]. These parasites cause high morbidity in school children and women during child-bearing age. The IPIs occur wherever there are poor living conditions, which immensely contribute to economic loss and poor health [1, 3, 4]. 

*Ascaris lumbricoides*, *Trichuris trichiura,* and hookworm species, collectively referred to as soil-transmitted helminths (STHs), are the most common intestinal parasites known to mankind [5]. Bethony et al. [5] have observed that children living in less developed countries are likely to be infected with one or more STH. Infections with these parasites affect the physical and cognitive development of school-age children [5]. Similarly, *Giardia lamblia* infects about 200 million people and is the most common parasitic protozoan flagellate worldwide [6, 7]. Like in other parts of the world [8], it has been documented that in western Kenya [9] the hookworm and other parasitic infections affect the haemoglobin and iron status of the children and adults. In the Republic of South Africa, Appleton et al. [10] have cautioned that the rate of reinfections with STH in urban slums is alarmingly high, which negates the benefits of treatment alone as a means of containing the parasitic infections among the endemic populations. 

In a survey that involved 390 participants in other parts of western Kenya, 76.2% had at least one STH, and it showed that hookworm infections were associated with lower hemoglobin, resulting in anaemia [11]. Nokes et al. [12] have also investigated the impacts of *T. trichiura* in school children of 9–12 years and found that expulsion of the worms resulted in a significant improvement in tests of auditory short-term memory and a highly significant improvement in retrieval of long-term information. 

The IPIs are common in rural western Kenya [9], but the prevalence of these parasites among children of five years and below has not been documented in the Webuye Health and Demographic Surveillance System (HDSS) area. The data we report was obtained from a community-based survey, and the participants were younger compared to those who have participated in most studies conducted in western Kenya [11]. However, based on reports from other studies [5, 9, 11], we expected the prevalence of IPIs to be high, and documenting this would help in planning intervention strategies. This study was initiated after our first round data collection at the Webuye HDSS [13] and the Webuye District Hospital annual report for 2008 [14], which showed intestinal parasites ranked high in the health facility records.

## 2. Materials and Methods

### 2.1. Study Site

**Figure 1:**
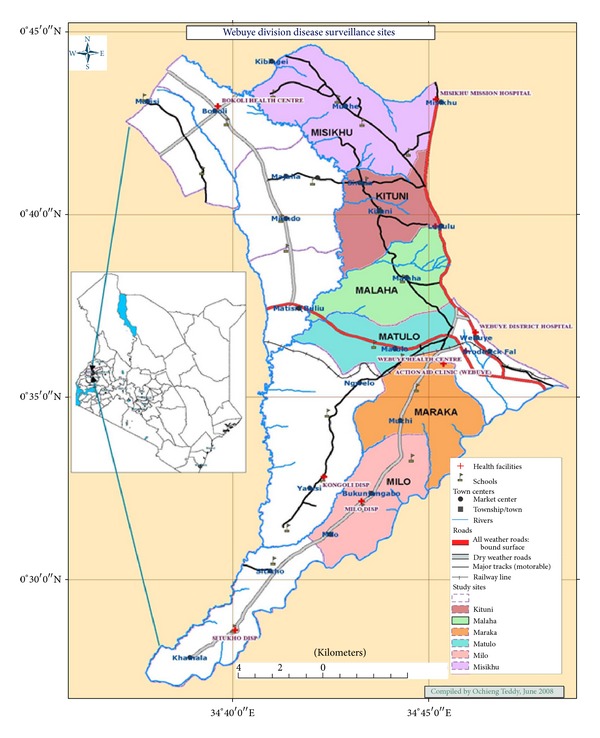
Webuye Health and Demographic Surveillance System area.

This survey was conducted at Webuye HDSS area (Figure 1), which is located at Webuye Division of Bungoma County, at an elevation of 1,523 m above sea level.The area has a tropical climate, and the land is mainly used for subsistence agriculture. It lies at a latitude of 0.6166667° and a longitude of 34.7666667°. During the study period, the temperatures ranged between 15 and 20°C to 23 and 30°C for minimum and maximum temperatures, respectively. The region receives high rainfall ranging from 1,200 mm to 1,800 mm annually. The first rains come in March to July, and the second one occurs from September to October [15]. The Webuye HDSS area comprises six sublocations in Webuye Division of Bungoma County, Kenya. The study site is exhaustively described elsewhere [16]. 

### 2.2. Study Population

The Webuye HDSS area has a well-defined population of about 80,000 people living in approximately 13,478 households in six sublocations within Webuye Division. The participants were selected from among children between 1 and 60 months who were captured in the Webuye HDSS database. 

### 2.3. Sample Size

This, being a prevalence study, the sample size was calculated using Fischer's formula as used by O. M. Mugenda and A. G. Mugenda [17], which is as follows: *n* = *z*_*α*_^2^ *p*(1 − *p*)/*d* ^2^, where *z* is the standard normal deviate = 1.96 for a 95% level of significance, *p* = 50% is the prevalence rate from other studies, and *d* = 0.04 is the degree of accuracy. Therefore, *n* = 1.96^2^0.5(1 − 0.5)/0.04^2^ = 600. To account for attrition/refusals/inability to produce stool specimen when required, we inflated the sample size by 30% and arrived at a minimum sample of size 780 of children who were recruited in the study.

### 2.4. Study Design, Sampling Frame and Sampling Techniques

Cross-sectional design was used to collect the data for this survey. Seven hundred and ninety seven (797) children were recruited from about 10,000 children of 5 years and below. Proportionate sampling was used to determine the number of children recruited from each sublocation. The village elders' compounds were used as the reference points in each village and an interval of 16 was used to identify subsequent compounds. The numbers of children recruited from each sublocation were as follows; 104, 132, 220, 122, 103, and 99 for Malaha, Matulo, Misikhu, Maraka, Milo, and Kituni, respectively, as shown in Table 1. If a child of the required age was not found in the selected compound, the next one where children of this age group were found and the head consented was recruited.

Only children of five years and below who were residents of Webuye and satisfied the criteria for inclusion in Webuye HDSS database were recruited. Joint Institutional Research and Ethics Committee (IREC) of the Moi Teaching and Referral Hospital (MTRH) and the School of Medicine, Moi University, Eldoret, approved the proposal of this project via clearance certificate no. IREC/2010/100 of 15/9/2010.

### 2.5. Stool Specimen Processing and Examination

This survey was conducted between September and December 2010. Only one specimen from each participating child was collected between 8.00 and 11.00 hours each day. The specimens were transported for processing and examination at the School of Medicine, Eldoret, between three to four hours after collection. Wet mounts were prepared from each specimen to determine the presence of intestinal protozoan trophozoites after which they were further processed using formal-ether-sedimentation technique previously described [18] to identify helminths ova and protozoan cysts. Briefly, a pie-size stool specimen was emulsified in 5 ml 10% formalin, and the preparation was strained through three layers of wet gauze. 4 ml diethyl ether was added and the mixture was shaken vigorously for about 30 seconds and centrifuged at 2000 rpm for about 5 minutes. The supernatant was decanted and the sediment dislodged using applicator stick. A portion of the preparation was transferred to a clean glass slide containing 1-2 drops of  1% Lugol's iodine. A cover glass was applied and the preparation was examined for ova and cysts. 

### 2.6. Data Handling and Analysis

The data for this survey were stored in an Excel spreadsheet, after which they were imported into SPSS version 12.5 database for statistical analysis. Descriptive statistics such as mean, median, standard deviation, and ranges were carried out for continuous data, while frequency listing and percentages were used to explore categorical data. To assess whether there was any association between the categorical variables, Pearson's Chi-square test was used. In all analyses, a *P* value less than 0.05 was considered significant.

## 3. Results and Discussion

### 3.1. Results

**Figure 2:**
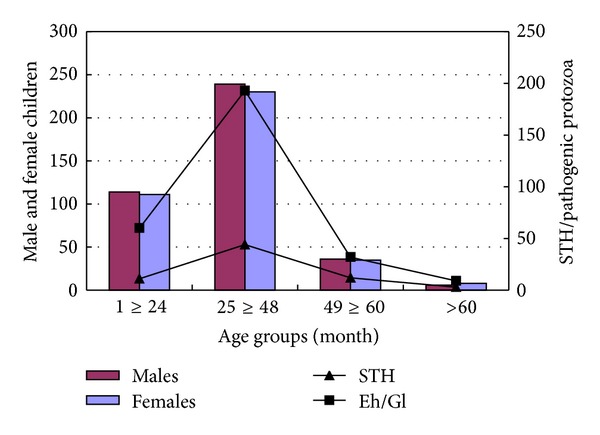
Soil-transmitted helminths (STHs) and pathogenic protozoan parasites among male and female children participants. STHs: soil-transmitted helminthes; Eh/Gl: *Entamoeba histolytica*/*Giardia lamblia. *

This cross-sectional survey recruited 797 children, of which 411 (51.6%) and 386 (48.4%) were boys and girls, respectively. Overall intestinal parasite prevalence of 52.3% (417/797) was obtained among children of five years and below in the Webuye HDSS. The male child was slightly more infected than the female in all age group categories, with greatest difference shown between 25 and 48 months age group (Figure 2). However, there were no significant differences in infections between male and female children (53% *versus* 51%; *χ* ^2^ = 0.482, *P* > 0.05). *Giardia lamblia* was the most common pathogenic intestinal protozoan flagellate with an overall prevalence of 26.1% (208/797) compared to *Entamoeba histolytica*, which was 11.2% (89/797). Among nonpathogenic intestinal protozoa found in all age groups, *Entamoeba coli* was the most prevalent (18.0%; 150/797). Other nonpathogenic protozoa identified included *Endolimax nana*, *Iodomoeba butschlii*, and *Chilomastix mesnili* which are nonpathogenic protozoa, were also identified (Table 1). Soil-transmitted helminths were comparatively less common with a prevalence of 4.8% (38/797), 3.8% (30/797), and 0.13% (1/797) for *Ascaris lumbricoides*, Hookworm species, and *Trichuris trichiura,* respectively. *Taenia* species was only encountered once among these rural children population (Table 2). The children participating in this survey were age stratified, and approximately 60% (476/797) were between the age group 24–48 months, out of which 74.4% (354/476) had either parasitic helminths (9.2%; 44/476) or protozoan infections (65.1%; 310/476). Infection with any parasites declined considerably after 48 months (Table 2). 

**Figure 3:**
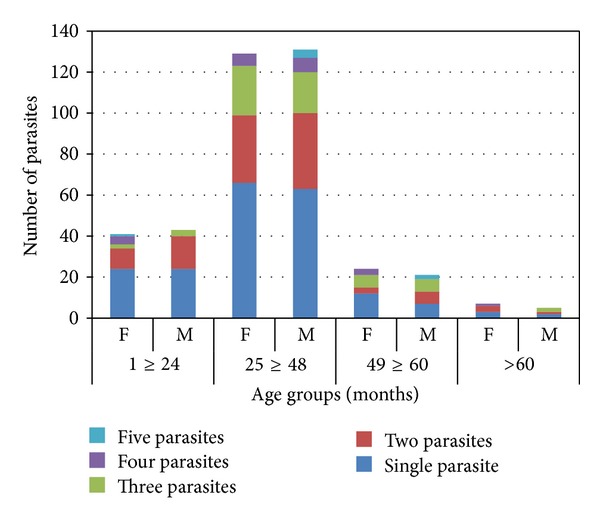
Patterns of single, double, and polyparasitisms among children participants in each age group.

Single and double parasitic infections per child were more prevalent (25.2%, 201/797; 13.7%, 109/797, resp.) compared to only 11.3% (91/797) polyparasitisms (≥3 parasites/individual) obtained among these rural children. Similarly, polyparasitisms were found more frequently in protozoa than in helminthic infections (Figure 3). Dual infections with STH were less comdmon, with only about 0.5% (4/797) children who had both *A. lumbricoides* and hookworm compared to 3.8% (30/797) children with *E. histolytica* and* G. lamblia* combination. The majority (2.6%; 20/797) of the children coinfected with *E. histolytica and G. lamblia* fall between ≥25 and 48 months age group (Table 3). 

### 3.2. Discussion

The prevalence of intestinal parasites found at Webuye HDSS area among children of five years and below was moderately high and was slightly higher amongst male children than the female. These results concur with those previously reported [19–22]. *Giardia lamblia* was the most common intestinal parasite among our study participants, which is also comparable to the results reported by Mehraj et al. [19], who found a similar trend in Karachi, Pakistan. Pillai and Kain [6] and Minenoa and Avery [7] have also showed that *G. lamblia* is the most prevalent protozoan parasite worldwide with about 200 million people infected. Mehraj et al. [19] attributed intestinal parasitic infections partly to poverty, illiteracy, poor hygiene, inaccessibility to potable water, and humid tropical climate. These conditions are somewhat similar to those found in Webuye HDSS area, where we conducted our study. This community has high accessibility to water; however, water obtained from these sources could be contaminated as shown by first-round Webuye HDSS data [13]. Furthermore, poverty incidence levels are high and are estimated between 56 and 60% in Bungoma County [15], which could predispose them to parasitic infection.

The present survey has shown that mixed infections within STH were less common, with only a few children found with both *A. lumbricoides* and hookworms compared to those coinfected with *E. histolytica* and* G. lamblia*. The majority (2.6%) of those who had mixed protozoan infections were children between ≥25 and 48 months. These children also had high IPIs infectivity rates. This age group fall within the period when children are increasingly involved in outdoor activities, including handling fecal contaminated materials, which predispose them to parasitic infections. Infection with any parasites declined considerably after 48 months partly because of the deworming program initiated by the Kenya Government's Ministry of Public Health and Sanitation, which target school-age children between 5–12 years old [23]. This program also targets the younger children, but with a few benefit because they require parental escort to access these services whenever it is available at the nearby primary schools. However, the parents are often engaged in other daily cores and would seldom remember the deworming schedule. In Pakistan, Chaudhry et al. [24] have also reported a decline in parasitic infection after the age of 2 years, but they did not offer any explanation for this. 

Single and double parasitic infections per child were more common compared to polyparasitism, probably due to the restricted transmission potential, which limited multiple infections among these rural children. Polyparasitism was common within the protozoa than in helminthic infections, but this was probably the outcome of a more hostile weather to the mode of transmission of helminths [25]. It is debatable whether polyparasitisms confer any advantage to those infected. According to a report by Jardim-Botelho et al. [26], children coinfected with *A. lumbricoides* and hookworms in Brazil had greater odds of poor performance in class than those infected with *A. lumbricoides* alone. Keusch and Migasena [27] have suggested possible synergistic effects of polyparasitisms, but this is inconclusive due to very few analyzed data.

Surprisingly, it appeared from our data that the prevalence of soil-transmitted helminths was low compared to the pathogenic protozoa such as *E. histolytica* and *G. lamblia*, suggesting that there could be factors mitigating low prevalence of STH at Webuye HDSS area. Furthermore, a similar survey involving women in a nearby rural western Kenya reported 76.2% infectivity rate with at least one geohelminth compared to our data (8.8%) [11]. This requires further investigations. However, accessibility of improved hygiene and sanitation and awareness of the diseases associated with lack of access to potable water could be one plausible explanation for low STH prevalence within this community [12, 13]. Access to shallow wells in Webuye HDSS area is moderately high (46.1%), which minimizes transmission potential of one member of STH group, hookworm species, which is transmitted by larval penetration of the skin in surface water [12, 25]. The transmission of the other two members of STH, *A. lumbricoides* and *T. trichiura,* is feco-oral requiring suitable environment for egg maturation, survival, and transmission. But this is partly hampered by the climate, which has a wet spell between September and December each year, during the period which temperatures may be conducive, but the type of soils found in Webuye is mostly clay enriched, which most probably hold on to the eggs. This type of soils also retains heat for long periods, which is inimical to the development and dispersion of helminths eggs [28, 29]. The children infected with intestinal parasites were treated at Webuye District Hospital and other nearby health facilities; however, helminths egg counts and anthropometric measurements were not undertaken to provide the baseline information upon which future studies can be assessed.

## 4. Conclusions and Recommendations

*Giardia lamblia* and *E. histolytica* were the most prevalent pathogenic intestinal protozoa, while STHs were less common among these rural children. Further research is required to elucidate the factors responsible for low STH prevalence among this rural children population. The parasites reported here are feco-orally transmitted and can easily be contained by the improvement of environmental hygiene. Community-based health promotions, including regular checkups and treatment and personal and environmental hygiene, are highly recommended for the control of the intestinal parasites infesting children at Webuye HDSS area.

## Figures and Tables

**Table 1:** The numbers of households allocated in each sublocation in Webuye HDSS.

Sublocation	Nos. of households	Sampling intervals	Targeted households
Malaha	1802	16	104
Matulo	2272	16	132
Misikhu	3792	16	220
Maraka	2115	16	122
Milo	1786	16	103
Kituni	1711	16	99

Total	13478	—	780

**Table 2:** Types and prevalence of intestinal parasites (percentages are in parentheses).

Variables	Female/male	Total		*P*-value
Gender distribution	*n* = 386	*n* = 411	*n* = 797	
All parasitic infections	197 (51.0)	220 (53.5)	417 (52.3)	0.482
*Ascaris lumbricoides *	19 (4.9)	19 (4.6)	38 (4.8)	0.843
Hookworm spp.	10 (2.6)	20 (4.9)	30 (3.8)	0.092
*Trichuris trichiura *	0	1 (0.2)	1 (0.13)	—
*Taenia* spp.	0	1 (0.2)	1 (0.13)	—
*Entamoeba histolytica *	35 (9.1)	51 (12.4)	86 (11.2)	0.129
*Giardia lamblia *	100 (25.9)	108 (26.3)	208 (26.1)	0.905
*Entamoeba coli *	76 (19.7)	74 (19.2)	150 (18.8)	0.543
*Endolimax nana *	74 (19.2)	55 (13.4)	129 (16.2)	0.027
*Chilomastix mesnili *	16 (4.2)	15 (3.7)	31 (3.8)	0.718
*Iodomoeba butschlii *	16 (4.2)	10 (2.4)	26 (3.3)	0.174

**Table 3:** Mixed infections of specific soil-transmitted helminths and pathogenic protozoa in each age group.

Age group	A/H	A/G	H/G	A/EH	G/EH	A/H/EH	A/G/EH	T/EH	H/G/EH	Total
1 ≥ 24 months	1	3	0	0	5	0	0	0	0	9
25 ≥ 48 months	2	2	5	2	20	1	1	1	0	34
49 ≥ 60 months	1	1	1	0	4	0	0	0	1	8
>60 months	0	0	0	0	1	0	0	0	1	2

Total	4	6	6	2	30	1	1	1	2	53

Key: A: *Ascaris* spp, H: Hookworm spp, G: *G. lamblia*, EH: *E. histolytica*, and T: *Taenia* spp.
